# Long-term evaluation of clinical success and safety of omadacycline in nontuberculous mycobacteria infections: a retrospective, multicenter cohort of real-world health outcomes

**DOI:** 10.1128/aac.00824-23

**Published:** 2023-09-28

**Authors:** Amer El Ghali, Taylor Morrisette, Sara Alosaimy, Kristen Lucas, Maria G. Tupayachi-Ortiz, Raaga Vemula, Carly Wadle, Julie V. Philley, Carlos Mejia-Chew, Yasir Hamad, Ryan W. Stevens, John D. Zeuli, Andrew J. Webb, Christina T. Fiske, Anahit Simonyan, Christo L. Cimino, Mehriban Mammadova, Virginia E. Umana, Rodrigo Hasbun, Saira Butt, Kyle C. Molina, Michael Thomas, Emily A. Kaip, Jeannette Bouchard, Tristan W. Gore, Catessa Howard, M. Gabriela Cabanilla, Dana J. Holger, Jeremy J. Frens, Melissa Barger, Aaron Ong, Keira A. Cohen, Michael J. Rybak

**Affiliations:** 1 Anti-Infective Research Laboratory, Department of Pharmacy Practice, Eugene Applebaum College of Pharmacy and Health Sciences, Wayne State University, Detroit, Michigan, USA; 2 Department of Clinical Pharmacy & Outcomes Sciences, Medical University of South Carolina College of Pharmacy, Charleston, South Carolina, USA; 3 Department of Pharmacy Services, Medical University of South Carolina (MUSC) Health, Charleston, South Carolina, USA; 4 Department of Medicine, Division of Pulmonary and Critical Care Medicine, Miller School of Medicine, University of Miami, Miami, Florida, USA; 5 University of Texas Health Science Center, University of Texas, Tyler, Texas, USA; 6 Division of Infectious Diseases, Department of Medicine, Washington University School of Medicine, St. Louis, Missouri, USA; 7 Department of Pharmacy, Mayo Clinic, Rochester, Minnesota, USA; 8 Division of Infectious Diseases, Department of Medicine, Vanderbilt University Medical Center, Nashville, Tennessee, USA; 9 Department of Pharmaceutical Services, Vanderbilt University Medical Center, Nashville, Tennessee, USA; 10 Division of Infectious Diseases, Department of Medicine, University of Texas Health Science Center at Houston, Houston, Texas, USA; 11 Division of Infectious Diseases, Indiana University School of Medicine, Indianapolis, Indiana, USA; 12 Department of Emergency Medicine, University of Colorado School of Medicine, Aurora, Colorado, USA; 13 Department of Pharmaceutical Services, University of California, San Francisco Medical Center, San Francisco, North Carolina, USA; 14 College of Pharmacy, University of South Carolina, Columbia, South Carolina, USA; 15 Department of Pharmacy, West Virginia University Medicine, Morgantown, West Virginia, USA; 16 Division of Infectious Diseases, University of New Mexico Health Sciences Center, Albuquerque, New Mexico, USA; 17 Department of Pharmacy Practice, Barry and Judy Silverman College of Pharmacy, Nova Southeastern University, Fort Lauderdale, Florida, USA; 18 Department of Pharmacy Services, Cone Health, Greensboro, North Carolina, USA; 19 Department of Medicine, Ventura County Medical Center, Ventura, California, USA; 20 Division of Pulmonary and Critical Care Medicine, Johns Hopkins University School of Medicine, Baltimore, Marlyand, USA; 21 Department of Medicine, Division of Infectious Diseases, School of Medicine, Wayne State University, Detroit, Michigan, USA; 22 Department of Pharmacy Services, Detroit Receiving Hospital, Detroit Medical Center, Detroit, Michigan, USA; Houston Methodist Academic Institute, Houston, Texas, USA

**Keywords:** omadacycline, nontuberculous mycobacteria, *Mycobacterium abscessus*, culture conversion

## Abstract

Infections due to nontuberculous mycobacteria (NTM) continue to increase in prevalence, leading to problematic clinical outcomes. Omadacycline (OMC) is an aminomethylcycline antibiotic with FDA orphan drug and fast-track designations for pulmonary NTM infections, including *Mycobacteroides abscessus* (MAB). This multicenter retrospective study across 16 U.S. medical institutions from January 2020 to March 2023 examined the long-term clinical success, safety, and tolerability of OMC for NTM infections. The cohort included patients aged ≥18 yr, who were clinically evaluable, and` had been treated with OMC for ≥3 mo without a previous diagnosis of cystic fibrosis. The primary outcome was 3 mo clinical success, with secondary outcomes including clinical improvement and mortality at 6- and 12 mo, persistence or reemergence of infection, adverse effects, and reasons for OMC utilization. Seventy-five patients were included in this analysis. Most patients were female (48/75, 64.0%) or Caucasian (58/75, 77.3%), with a median (IQR) age of 59 yr (49–67). Most had NTM pulmonary disease (33/75, 44.0%), skin and soft tissue disease (19/75, 25.3%), or osteomyelitis (10/75, 13.3%), and *Mycobacterium abscessus* (60/75, 80%) was the most commonly isolated NTM pathogen. The median (IQR) treatment duration was 6 mo (4 – 14), and the most commonly co-administered antibiotic was azithromycin (33/70, 47.1%). Three-month clinical success was observed in 80.0% (60/75) of patients, and AEs attributable to OMC occurred in 32.0% (24/75) of patients, leading to drug discontinuation in 9.3% (7/75).

## INTRODUCTION

*Mycobacteroides abscessus* (MAB), previously known as *Mycobacterium abscessus*, represents a specific group of environmental pathogens within the nontuberculous mycobacteria (NTM) family that pose significant treatment challenges (1, 2). The incidence of MAB infection is increasing in the United States and has been reported to be among the most frequently isolated NTM pathogens in patients with NTM pulmonary disease (NTM-PD) (3 – 6). Infections caused by these pathogens are notoriously difficult to manage because of factors such as slow growth rate, multiple intrinsic resistance mechanisms, and biofilm formation (2, 7). This often requires regimens involving several antibiotics that are frequently administered parenterally for extended durations of therapy, commonly resulting in logistical issues, patient intolerance, and adherence concerns (2). The increasing prevalence of MAB, which exhibits high levels of intrinsic macrolide resistance, poses a significant challenge for treatment strategies. A functional *erm* gene, which can be found in MAB, confer inducible macrolide resistance. A recent study found that over 70% (426/607) of tested MAB subspecies *abscessus* isolates had a functional *erm* gene (8). Furthermore, macrolide resistance significantly affects clinical outcomes, as only 25% of cases of macrolide-resistant MAB-associated pulmonary disease attained sustained sputum conversion after 1 yr (9). The clinical consequences of macrolide resistance are substantial and could render treatment more complicated. Given that macrolides are accessible in oral form, providing a unique advantage in terms of prescribing and patient acceptance, they are often deemed a fundamental aspect of treatment (2). However, the prospect of their constrained use, owing to escalating resistance, introduces an added dimension of complexity in the management of mycobacterial disease.

This complexity is further amplified by the increasing prevalence of MAB and MAB-associated pulmonary diseases (4 – 6). The resilience of this pathogen to conventional antibiotics, coupled with the necessity for protracted therapy and the lack of effective oral treatment alternatives, highlights the pressing need for the development of new orally administrable therapies that are active against this pathogen (3, 6).

Omadacycline (OMC) is a novel first-in-class aminomethylcycline that provides broad-spectrum antimicrobial coverage and is available in both oral and intravenous formulations. It has received approval from the U.S. Food and Drug Administration (FDA) for the treatment of adults with community-acquired bacterial pneumonia (CABP) or acute bacterial skin and soft tissue infections (ABSSI) (10). Both *in vitro* and *in vivo* studies have demonstrated OMC’s potency and effectiveness against *M. abscessus, Mycobacteroides chelonae (M. chelonae) ,* and *Mycobacterium fortuitum (M. fortuitim)* infections (11 – 15). Similar to its tetracycline counterparts, OMC functions by binding to the 30S ribosomal subunit, thereby inhibiting protein synthesis (16). However, unlike first-generation tetracyclines, OMC has been deliberately engineered to evade ribosomal protection and tetracycline efflux mechanisms. In the context of MAB, the production of a monooxygenase enzyme may degrade tetracyclines, such as minocycline and doxycycline, but does not affect OMC (17). This feature, in part, revitalizes its activity against recalcitrant MAB and may serve as a deterrent against the development of resistance during treatment (16). Moreover, intolerance during treatment is a frequent reason for drug cessation, especially with tetracyclines, where symptoms such as elevated liver enzymes and gastrointestinal discomfort often necessitate discontinuation (18, 19). Emerging evidence suggests that OMC may be more tolerable and potentially less likely than tigecycline (TIG) to cause these issues (10, 18 – 21).

OMC has recieved orphan drug status and fast-track designation by the FDA for pulmonary NTM infections and is currently being tested in a phase II trial (ClinicalTrials.gov Identifier: NCT04922554)(22). Despite the increasing use of OMC in the treatment of NTM infections,, there is a paucity of real-world data regarding its outcomes, safety, and treatment-specific factors. Such information can significantly aid clinicians in making informed decisions regarding patient care. Therefore, the primary objective of this study is to evaluate the long-term effectiveness, safety, and tolerability of OMC treatment in patients with both pulmonary and extrapulmonary NTM infections.

## MATERIALS AND METHODS

This was a retrospective, cohort study involving 16 U.S. academic medical centers from 1 January 2020 to 30 March 2023. Patients were included if they were ≥18 yr of age, received OMC in any dosage form for≥72h, and were clinically evaluable for≥3 mo with a documented follow-up. Patients were excluded if they were pregnant or nursing mothers, prisoners, or had cystic fibrosis. Any subsequent OMC courses were included only if they were separated by≥60 d. Clinically evaluable durations started from the time of initiation of OMC treatment. The primary outcome was a 3 mo clinical success, defined as a composite of survival, clinician-evaluated clinical improvement at 3 mo, lack of alteration in OMC therapy due to treatment failure or AE, and lack of microbiological recurrence. Secondary outcomes included sputum culture conversion at 12 mo and 6- and 12 mo clinical success when available (composites of the components mentioned previously), 3-, 6-, and 12 mo imaging improvement (when applicable), AE, and reasons for OMC utilization.

Culture conversion was defined as ≥2 consecutive negative sputum cultures within 12 mo of OMC initiation, with no further positive cultures, each taken at least 1 mo apart. This definition was applied only to patients who had positive cultures upon the start of OMC therapy. Microbiological recurrence was defined as≥2 consecutive positive cultures for the same pathogen isolated from the index culture following sputum culture conversion (respiratory) or microbiological clearance (non-respiratory). Dissemination was defined as the isolation of the NTM pathogen in ≥2 separate organ sites. Clinical and Laboratory Standards Institute (CLSI) susceptibility breakpoints were applied (when applicable) to interpret mean inhibitory concentration (MIC) values, whereas FDA antibacterial susceptibility interpretive criteria were used when information was unavailable via CLSI (23, 24). We characterized combination therapy as the co-administration of any antibiotic with OMC for >28 d. While all adverse effects of OMC have been recorded, it is important to mention that they cannot be exclusively ascribed to OMC because of the concurrent use of other treatments. Descriptive statistics were used for analyses. Chi-square or Fisher’s exact tests were used for categorical data, and either student’s *t*-test or Mann Whitney U test was used for continuous variables. Bivariate analysis was used to identify the characteristics potentially associated with clinical or safety outcomes. Statistical significance was set at *P* < 0.05. Statistical analysis was performed using the IBM SPSS software (version 29.0; SPSS, Inc., Chicago, IL, USA). Our study, due to its retrospective design, fell under the category of research exempt from Institutional Review Board (IRB) review. As we only analyzed pre-existing, de-identified data, there was no need for direct patient contact or further consent.

## RESULTS

### Patient baseline characteristics

In total, 75 patients met the inclusion criteria, while eight patien were excluded due to insufficient 3 mo OMC exposure or follow-up. The included patients were predominantly female (48/75, 64.0%) and Caucasian (58/75, 77.3%). These patients had a median age [interquartile range (IQR)] of 59 (49–67) yr, a median (IQR) body mass index at baseline of 25.28 (21.2–30.8) kg/m^2^ and 9.3% (7/75) were classified as underweight, with a BMI<18.5kg/m^2^ (Table 1). Most patients were treated strictly in the outpatient setting (53/75, 70.6%), with 26.6% (20/75) receiving OMC initiated in the hospital and continuing therapy in the outpatient setting. Of the patients admitted to the hospital, 50% (10/20) were admitted to the ICU≥1time. The mean [standard deviation (SD)] Charlson Comorbidity Index was 3.1 (2.2), with the most common comorbidities being asthma (13/75, 17.3%), heart failure (10/75, 13.3%), diabetes mellitus (10/75, 13.3%), osteoarthritis (9/75, 12.0%), and chronic obstructive pulmonary disease (8/75, 10.7%). Notably, none of the patients had liver dysfunction at baseline, 6.7% (5/75) had acute kidney injury, and 6.7% (5/75) had moderate-to-severe chronic kidney disease (CKD). Additionally, 28% (21/75) had immunosuppressive conditions that included solid organ transplantation (4/75, 5.3%), hypogammaglobulinemia (4/75, 5.3%), high-dose oral corticosteroids (3/75, 4.0%), cytotoxic chemotherapy (2/75, 2.7%), splenectomy (2/75, 2.7%), and other (6/75, 8.0%).

**TABLE 1:** Patient baseline characteristics^*a*^

Characteristics	(*n* = 75)
Age, yr, median (IQR)	59 (49–67)
Female	48 (64.0)
BMI, kg/m², median (IQR)	25 (21–31)
BMI ≥ 30 kg/m² (obese)	21 (28.0)
BMI <18.5 kg/m² (underweight)	7 (9.3)
Caucasian	58 (77.3)
Treatment setting	----
Strictly outpatient	53 (70.7)
Inpatient, then outpatient	22 (29.3)
Insurance	----
Government	34 (45.3)
Private	32 (42.7)
Mixed (Government and private)	9 (12.0)
Charlson Comorbidity Index, mean (SD)	3.1 (2.2)
Comorbid conditions	----
None	22 (29.3)
Immunosuppression	18 (24.0)
Asthma	13 (17.3)
Diabetes (type II)	10 (13.3)
Heart failure	10 (13.3)
Connective tissue disease (e.g., osteoarthritis, rheumatoid arthritis, etc.)	9 (12.0)
Chronic obstructive pulmonary disease (COPD)	8 (10.7)
Interstitial lung disease (pulmonary fibrosis)	7 (9.3)
Autoimmune disease	7 (11.1)

^*a*^ Data reported as *n* (%) unless otherwise specified.

### Infection characteristics

The infection characteristics are shown in Table 2. The majority of the infections were NTM-PD (33/75, 44.0%), skin and soft tissue (19/75, 25.3%), and osteomyelitis (10/75, 13.3%). Radiographic patterns of nodular/bronchiectasis (tree-in-bud, bronchonodular, and cluster of micronodular) and cavitary disease associated with NTM-PD were present in 81.8% (27/33) and 18.2% (6/33) of the patients, respectively. When examining the clinical criteria for NTM-PD (chronic cough, hemoptysis, fatigue, weight loss>5%, and/or night sweats), 60.6% (20/33) had 1–2 symptoms, 39.4% (13/33) had≥3 symptoms. Most patients had either chronic cough (26/33, 81.8%) and/or fatigue (21/33, 63.6).

**TABLE 2:** Treatment characteristics^*c*^

Characteristics	(*n* = 75)
Infection source	----
Pulmonary disease	33 (44)
Skin and soft tissue	17 (27.4)
Osteomyelitis	10 (16.1)
Invasive prosthetic device (LVAD, etc.)	5 (6.7)
Intra-abdominal	3 (4.0)
Other^*a*^	5 (6.7)
NTM species	
*M. abscessus*	60 (80.0)
*M. chelonae*	9 (12.0)
*M. avium* complex	7 (9.3)
*M. fortuitum*	4 (5.3)
Subspeciation performed (MAB)	35 (58.3)
Subspecies *abscessus*	25 (71.4)
Subspecies *massilliense*	9 (25.7)
Subspecies *bolletti*	1 (2.9)
e*rm* gene testing performed (MAB)	27 (45.0)
*erm* gene functional	26 (96.3)
Polymicrobial infection (Gram-negative, Gram-positive)	26 (35.0)
NTM-PD symptom criteria^*b*^ (*n* = 33)	
Chronic cough	26 (34.7)
Fatigue	20 (26.7)
Weight loss (>5%)	9 (12.0)
Hemoptysis	8 (10.7)
Night sweats	5 (6.7)
Number of symptoms (*n* = 33)	----
1–2	20 (60.6)
3–5	13 (39.4)
Radiologic findings (*n* = 33)	----
Nodular	11 (33.3)
Tree-in-bud	11 (33.3)
Bronchonodular	9 (27.2)
Fibrocavitary	6 (18.2)
OMC duration, mo, median (IQR)	6 (4 – 14)
OMC follow-up, mo, median (IQR)	7 (3–12.5)
Treatment type	----
Targeted	56 (74.7)
Suppression	17 (22.7)
Empiric	4 (5.3)
Dissemination (prior to OMC initiation)^*d*^	8 (10.7)
OMC loading dose	25 (33.3)
450 mg PO once daily, 1st 2 d	18 (24.0)
Other	7 (9.3)
OMC MIC, μg/mL, median (IQR) (*n* = 10)	0.5 (0.22–0.63)
TIG MIC, μg/mL, median (IQR) (*n* = 61)	0.25 (0.12–0.5)
Reasons for OMC use	
Ease of administration	46 (61.3)
Oral bioavailability	41 (54.7)
Resistance to other agents	34 (45.3)
Previous antibiotic failure	22 (29.3)
Concomitant antibiotic(s) with OMC	70 (93.3)
Azithromycin	33 (44.0)
Clofazimine	29 (38.7)
Linezolid/tedizolid	24 (32.0)
Imipenem (I.V.)^*e*^	22 (29.3)
Amikacin (I.V.)	19 (25.3)
Amikacin (inhaled)	16 (21.4)

^*a*^ Other infection sources were as follows otitis media (*n*=2), infective endocarditis (*n*=1), central line (*n*=1), external ventricular drain (*n*=1).

^*b*^ NTM Symptom Criteria: Chronic cough, night sweats, weight loss >5%, hemoptysis, fatigue NTM, Nontuberculous mycobacteria.

^*c*^ Data reported as *n* (%), unless otherwise specified; Patients received oral OMC 300 mg.

^*d*^ OMC, omadacycline.

^*e*^ I.V, intravenous.

Among patients with MAB-associated skin and soft tissue infections, 52.6% (10/19) developed a surgical wound infection, 42.1% (8/19) presented with an abscess, and 15.8% (3/19) presented with an ulcer. Patients with NTM-associated osteomyelitis had a primary infection in the foot (4/10, 40%), hip (2/10, 20%), vertebrae (2/10, 20%), hand (1/10, 10%), and knee (1/10, 10%), with an equal distribution of acute (5/10, 50%) and chronic (5/10, 50%) infections. Three of the 75 patients had NTM-associated intra-abdominal infections, with 2/3 having multiple abscesses and 1/3 having a single abscess. All three intra-abdominal infections were related to a surgical procedure. One patient had NTM-associated infective endocarditis secondary to cardiac prosthesis.

**Fig 1:**
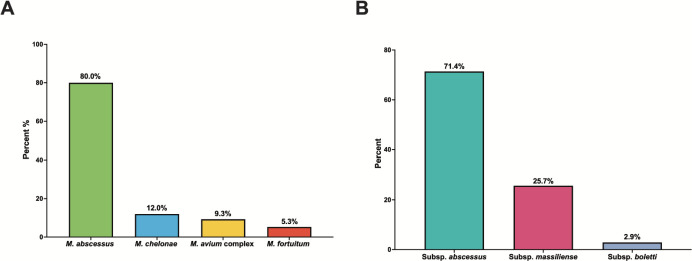
Mycobacterium species characteristics. (A) *n* = 75, Breakdown of all mycobacteria isolated from cultures; NTM, nontuberculous mycobacterium (B) *n* = 35, Characterization of subspeciation results of patients where data were available; Subsp., subspecies.

Most treated NTM isolates were MAB (60/75, 80%); however, 12% (9/75) were *M. chelonae*, 9.3% (7/75) were *Mycobacterium avium* complex (MAC), and 5.3% (4/75) were *M. fortuitum* (Fig. 1). Notably, 12% (9/75) of patients had positive cultures with two or more of the four aforementioned NTM species and 6/9 were sputum cultures (6/6, MAC; 1/6, *M. fortuitum*), and all patients with MAC had a concomitant MAB infection. In patients with a MAB-associated infection, subspeciation was performed in 58.3% (35/60), with the majority being *M. abscessus* subsp. *abscessus* (25/35, 71.4%), whereas subsp. *massilliense* and subsp. *boletti* were present in (9/35, 25.7%) and (1/35, 2.9%) patients, respectively (Fig. 1). The *erm* gene, which is responsible for inducible target site modification of macrolides and thus increased macrolide resistance, was tested in 45.0% (27/60) of patients with MAB, and 96.3% (26/27) of the tested isolates exhibited functional *erm* gene expression (25). Thirty-five percent (26/75) of patients had a variety of gram-negative and gram-positive pathogens isolated concomitantly from the primary infection site, with 57.6% (19/33) of patients with NTM-PD having multimicrobial cultures present. The most common co-isolated bacteria were *Pseudomonas aeruginosa* (11/26, 42.3%)*, Aspergillus* spp. (7/26, 26.9%), *Stenotrophomonas maltophilia* (5/26, 19.2%), and *Achromobacter xylosoxidans* (5/26, 19.2%); all were isolated from sputum.

Before OMC initiation, antibiotic therapy was administered in 84% (63/75) of patients for a median (IQR) duration of 5.1 (3.6–13.2) mo. OMC was predominantly used as a targeted therapy (60/75, 80%), while it was also used for suppressive and empiric therapy in 22.7% (17/75) and 5.3% (4/75) of patients, respectively. Dissemination of NTM was observed in 10.7% (8/75) of patients prior to OMC initiation. The primary reasons for the use of OMC were ease of administration (48/75, 64%), oral bioavailability (41/75, 54.7%), resistance to other agents (34/75, 45.3%), and previous antibiotic failure (22/75, 32%). Positive NTM cultures at the time of OMC initiation were present in 70% (39/56) of patients for whom this information was known. Only 13.3% (10/75) of the isolates underwent Mean Inhibitory Concentrations (MIC) testing for OMC (range, 0.06–1.0; MIC_90,_ 1 µg/mL). However, tigecycline (TIG) MIC values were reported in 80% (60/75) of isolates (range, 0.03–4.0 µg/mL, MIC_90,_ 1 µg/mL), with further MIC distributions listed in Table 3.

**TABLE 3:** Distribution of mean inhibitory concentrations for *M. abscessus* species^*a*^

Antibiotic	MIC_50_ (mcg/mL)	MIC_90_ (mcg/mL)	Range (mcg/mL)
Omadacycline (*n* = 11)	0.5	1	0.06–1
Tigecycline (*n* = 49)	0.25	1	0.03–4
Amikacin (*n* = 49)	16	32	8–128
Azithromycin (*n* = 14)	32	256	≤16–>256
Clofazimine (*n* = 28)	0.5	0.5	0.03–0.5
Imipenem (*n* = 49)	16	16	≤2–>32
Linezolid (*n* = 47)	16	32	0.5–64

^*a*^ Distribution of available MIC data for all *Mycobacteroides abscessus* species. Some MICs were listed as greater than or equal to and the lesser value was utilized in calculation of the MIC_50_ and MIC_90_ values.

**Fig 2:**
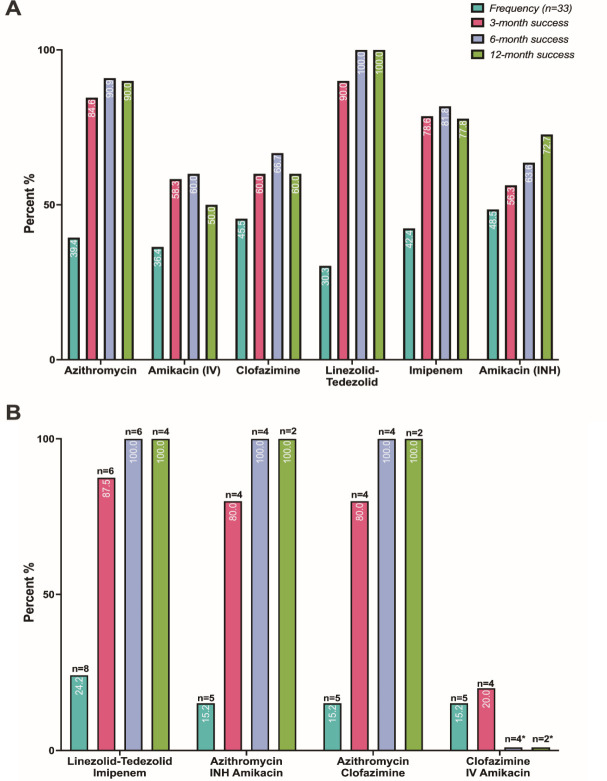
Outcomes of concomitantly administered antibiotics with omadacycline. (A) Frequency and clinical outcomes associated with combined antibiotics with omadacycline in patients with nontuberculous Mycobacterium-pulmonary disease (NTM-PD) represents outcomes for any regimen containing the individual antibiotic; IV, intravenous; INH, inhaled (B) Frequency and clinical outcomes of the most utilized drug regimens combined with OMC in NTM-PD patients (*n* = 33); INH, inhaled; IV, intravenous; * Did not meet clinical success endpoint.

The total median duration of OMC (IQR) was 6.2 (3.7–14.6) mo, whereas the median duration of follow-up after OMC initiation (IQR) was 7 (3–12.5) mo. Most patients received ≥2 antibiotics combined with OMC (70/75, 93.3%), with azithromycin (AZM, 33/70, 47.1%), clofazimine (CFZ, 29/70, 41.4%), linezolid-tedizolid (LNZ-TED, 24/70, 34.2%), imipenem (IMI, 22/70, 31.4%), IV amikacin (IV-AMK, 19/70, 27.1%), and/or inhaled amikacin (INH-AMK, 16/70, 22.9%) being the most common co-administered antibiotics. Interestingly, five patients received OMC as monotherapy (1-pneumonia, 1-osteomyelitis, 1-skin and soft tissue, 1-otitis media, and 1-external ventricular device). When examining patients with NTM-PD, the most frequently used OMC-containing treatment regimens were OMC/LNZ-TED/IMI (8/33, 24.2%), OMC/AZM/INH-AMK (5/33, 15.2%), OMC/AZM/CFZ (5/33, 15.2%), and OMC/CFZ/IV-AMK (5/33, 15.2%) (Fig. 2). Oral OMC was administered to all patients. Of these, 88.0% (66/75) received a maintenance dose of 300 mg/day, whereas 9.3% (7/75) received a daily dose of 150 mg. Additionally, 24% (18/75) of the patients were administered an initial oral loading dose of 450 mg/day for the first 2 d.

### Clinical outcomes

Three-month composite clinical success was observed in 80.0% (60/75) of patients (Table 4). Of the 15/75 patients who did not achieve clinical success, 10.7% (8/75) had 30 d microbiological recurrence, 5.3% (4/75) did not show clinician-evaluated clinical improvement, and 4.0% (3/75) switched from OMC for suspected therapeutic failure or AE. For patients for whom data were available, the composite clinical success rates at 6- and 12 mo were 78.7% (48/61) and 76.0% (41/54), respectively. Among patients with NTM-PD who underwent radiological follow-up after OMC initiation, 3-, 6-, and 12 mo imaging improvements were observed in 60% (18/30), 64.3% (18/28), and 68.2% (15/22) of patients, respectively. Among patients who had a functional *erm* gene present, 76.9% (20/26), 85.7% (17/21), and 77.8% (14/18) met the 3-, 6-, and 12 mo clinical success endpoints, respectively. Three patients died (3/75, 4%) during the treatment period, one within 6 mo and two within 12 mo of starting OMC. Only one death was considered infection-related, whereas the other two deaths were related to comorbid conditions. Of the 28 patients with NTM-PD who underwent follow-up cultures, 79% (22/28) achieved culture conversion and the median time to culture conversion (IQR) was 5 (2–11.5) mo. When examining patients with NTM-PD, 3-, 6-, and 12 mo composite clinical success was observed in 66.7% (22/33), 70.3% (19/27), and 66.7% (16/24) of patients, respectively. When assessing the effectiveness of various combinations of antibiotics and treatment plans, regimens incorporating AZT, LZD/TED, and IMI yielded the best clinical outcomes when used in conjunction with OMC. Conversely, OMC regimens that included either inhaled or intravenous AMK appeared less effective (Fig. 2). The unadjusted factors associated with clinical failure at 3 mo were NTM-PD (*P* = 0.011), nodular bronchiectasis (*P* = 0.007), and chronic cough at baseline (*P* = 0.035) (Table 5).

**TABLE 4:** Clinical outcomes

Outcomes	3 mo (*n* = 75)	6 mo (*n* = 61)	12 mo (*n* = 54)
Clinical success	60 (80.0)	48 (78.7)	41 (76.0)
Absence of microbiological recurrence	67 (89.3)	55 (80.2)	47 (87.0)
Clinician evaluated clinical improvement	66 (88.0)	56 (91.8)	47 (87.0)
Omadacycline continuity (no switch for failure/AE)	72 (96.0)	58 (95.1)	50 (92.6)
Survival	75 (100.0)	60 (98.4)	52 (96.3)
Imaging improvement	**(*n* = 30)**	**(*n* = 28)**	**(*n* = 22)**
	18 (60.0)	18 (64.3)	15 (68.2)
Culture conversion (≥2 consecutive negative cultures) (*n* = 52)	43 (82.7)		
Time to culture conversion, median (IQR), mo	5 (2–11.5)		

^*a*^ Data reported as *n* (%), unless otherwise specified; OMC, omadacycline; AE, adverse event; IQR, interquartile range.

**TABLE 5:** Bivariate analysis of factors potentially associated with 3 mo clinical failure^*a*^

Characteristic	All patients (*n* = 75)	Success (*n* = 60)	Failure (*n* = 15)	*P*-value (<0.05)
NTM-PD	33 (44.0)	22 (36.7)	11 (73.3)	0.011
Nodular/bronchiectasis	16 (21.3)	9 (15.0)	7 (46.7)	0.007
Chronic cough	26 (34.7)	17 (28.3)	9 (60.0)	0.021

^*a*^ Data reported as *n* (%), unless otherwise specified; All bivariate analysis was either conducted by Chi-square or Fisher’s Exact Test for categorical variables or Student’s t-test/Mann-Whitney U for continuous variables, P <0.05 was considered significant; All covariates were tested, but only covariates with a *P* <0.05 were included in this table; OMC, omadacycline; NTM-PD, nontuberculous mycobacteria pulmonary disease.

### Safety outcomes

Given the protracted nature of therapy for NTM, it is critical to understand the safety and tolerability of OMC. Tetracycline and tetracycline derivatives are known to cause AEs that lead to patient intolerance, particularly over extended durations. AEs were experienced by 32% (24/75) of patients, 9.3% (7/75) discontinued OMC due to intolerance, and 30.7% (23/75) had AE resolution while continuing on OMC (Table 6). Most AEs were gastrointestinal (nausea, vomiting, and diarrhea) (21/75, 28%) or hepatotoxic (AST/ALT elevation 3 × the upper limit of normal) (6/75, 8.0%) in nature. Notably, no specific factor was associated with the occurrence of AEs, and the median (IQR) time to gastrointestinal AE was 28 (10–88) d.

**TABLE 6:** Safety outcomes^*a*^

Safety outcomes	(*n* = 75)
Adverse events (AE)	24 (32.0)
Gastrointestinal (nausea, vomiting, diarrhea)	21 (28.0)
AST/ALT elevation (>3 × upper limit of normal)	6 (8.0)
Rash/dermatological reaction	2 (2.7)
Headache	2 (2.7)
Neutropenia	2 (2.7)
AE led to drug discontinuation (*n* = 24)	7 (9.3)
AE resolved (*n* = 24)	17 (22.7)
AE ongoing (*n* = 24)	3 (8.0)
AE resolved with residual effects (*n* = 24)	3 (4.0)
Time to GI related adverse event, days, median (IQR)	28 (10–88)

^*a*^ Data reported as *n* (%), unless otherwise specified; OMC, omadacycline.

## DISCUSSION

To our knowledge, this is one of the largest multicenter observational studies of its kind. Our investigation provides an in-depth examination of the long-term clinical success, tolerability, and safety of OMC for the treatment of both pulmonary and extrapulmonary NTM infections. Our findings revealed consistent rates of clinical success, which persisted in patients undergoing clinical follow-up. These outcomes not only reinforce the potential of OMC as a viable treatment approach for NTM infections but also align with the limited but existing data on the clinical success, safety, and tolerability of OMC (26– – 31 31 – 31). Although our study showed a greater incidence of gastrointestinal (28%) and hepatotoxic (8.0%) AEs, the overall AE rates were consistent with those reported in previous studies (26 – 31). Importantly, most previous studies utilized intravenous OMC, whereas in this study, all patients received the medication orally. As a result, a comparison of the AEs from the OASIS-2 trial, which evaluated once-daily oral OMC 300mg for ABSSI, may be the most appropriate (31). In our patient population, we reported overall AEs at a rate of 32.0% compared to 54.0% in the OASIS-2 trial. The occurrences of gastrointestinal and hepatotoxic AEs was also different, at 28.0% and 8.0% in our study compared to 37.8% and 10.0% in the OASIS-2 trial, respectively. The authors of the OASIS-2 trial noted that gastrointestinal AEs typically surfaced within the initial 1–2 d, potentially due to the use of loading doses. This may explain the observed diferences in our study, as only 33.0% (25/75) received any type of loading dose. We observed that patients who received oral loading doses had a higher incidence of gastrointestinal AEs (7/18, 38.9%) than those who did not (12/50, 24.0%). Given the concern for GI intolerance, it may be prudent to prescribe antiemetic medication on initiation to increase tolerability (21). Additionally, we attributed the increased frequency of GI AEs primarily to longer treatment durations, which typically manifested around the 28th day. Despite these concerns, we observed a low OMC discontinuation rate of 9.0%, proving that most AEs were transient in nature.

Thus, the importance of treatment tolerability in the management of NTM infections cannot be overstated. Although other tetracyclines, such as TIG and eravacycline (ERV), have shown substantial *in vitro* activity against NTM, their application has been restricted because of their parenteral-only formulations (ERV and TIG) and their high incidence of side effects (TIG) (18 – 20, 32). Interestingly, our study detected no discernible relationship between patient baseline or treatment characteristics and the incidence of AEs. However, we did observe that patients who received a loading dose did have a higher proportion of GI AE (*P* > 0.05). Furthermore, the once-daily oral administration of OMC and higher reported sustained lung penetration may offer an additional advantage over TIG (20, 33). Patient adherence and preference for oral medications emerge as crucial considerations, given the need for long-term NTM treatmen. These factors were key motivators for prescribers’ use of OMC in our study. Compared with twice-daily IV alternatives, a once-daily oral regimen could potentially reduce the risk of patient non-adherence and increased costs associated with outpatient parental therapy (34, 35). In terms of treatment outcomes, our study observed high culture conversion rates at 12 mo in patients who underwent follow-up cultures, further supporting our composite clinical success outcomes. Although *M. abscessus* was the primary pathogen isolated during treatment, we also encountered patients with *M. avium* complex, *M. fortuitum*, and *M. chelonae* infections. Several of these pathogens were isolated concomitantly with *M. abscessus* which may have complicated treatment.

The intrinsic and inducible resistance found in MAB may serve as an area where OMC could become a viable treatment option. Our study found similar clinical success rates, regardless of the presence of a functional *erm* gene, which highlights the potential of OMC in situations where macrolide resistance or the presence of the *erm* gene is suspected or detected. Unlike macrolides, OMC is not influenced by functional *erm* and is less prone to ribosomal protection and efflux pumps, which are common resistance mechanisms that affect many antibiotics, including tetracyclines. Finally, our bivariate analysis helped to identify factors potentially linked to treatment failure. Not surprisingly, patients with NTM-PD, chronic cough, and bronchiectasis had significantly higher rates of clinical failure.

While these findings offer valuable insights, our study has several limitations. First, the retrospective design may have introduced a selection bias, and the lack of standardization in treatment and follow-up duration could have influenced the results. Second, the inclusion of both pulmonary and extrapulmonary NTM infections in the analysis may complicate comparisons, particularly given the importance of source control in the management of extrapulmonary NTM. Third, the limited availability of susceptibility data for OMC underlines the inadequate application of existing antimicrobial susceptibility testing (AST) techniques for OMC. This emphasizes the importance of integrating OMC into testing panels or examining the possibility of employing TIG MICs as a surrogate for evaluating OMC susceptibility. Lastly, reliance on clinician-evaluated clinical improvement without predefined criteria may lead to variability in interpretation and potential ambiguity depending on the clinician.

In conclusion, despite its limitations, this study provides a realistic representation of OMC use in outpatient clinical settings, where data completeness is often challenging. The practical outcomes and insights from this research can offer significant value to clinicians in their daily practice of treating these infections and shed light on the long-term outcomes and safety profile of OMC to treat NTM infections, an area where existing data are notably sparse. Phase II trials of OMC for the treatment of NTM infections are currently in progress. However, there is still a need for additional data to fully establish the therapeutic potential and role of OMC. This should include studies examining cost-effectiveness in comparison to intravenous treatments, evaluations of outcomes in patients with cystic fibrosis, and translational research that bridges in vitro data with clinical results.
